# Effects of two-dimensional cyber incivility on employee well-being from a self-determination perspective

**DOI:** 10.3389/fpsyg.2023.1137587

**Published:** 2023-04-11

**Authors:** Shuai-Ping Xiao, Yi Lu, Yu Yan, Zhiqing E. Zhou, Zhao-Xue Cao, Kai-Chen Zhao

**Affiliations:** ^1^Department of Psychology, School of Philosophy, Wuhan University, Wuhan, China; ^2^Department of Psychology, Baruch College and The Graduate Center, City University of New York, New York, NY, United States

**Keywords:** cyber incivility, active, passive, emotional exhaustion, intrinsic motivation, promotion focus

## Abstract

The research attempts to explore the effects of two-dimensional cyber incivility on employee well-being. Based on self-determination theory and regulatory focus theory, we conducted two studies to examine the mediating role of intrinsic motivation and the moderating role of promotion focus between cyber incivility and emotional exhaustion. The results demonstrated that both active and passive cyber incivility predicted increased emotional exhaustion, with intrinsic motivation serving as a key mediator. There was no consistent conclusion of promotion focus’s moderating role. High promotion focus might aggravate the negative effect of passive cyber incivility on intrinsic motivation. The present article provides deeper step towards understanding of cyber incivility, which also helps in the development of intervention strategies to lessen or avoid the negative impact of work-related stressful events on employee well-being.

## 1. Introduction

The progress of information and communication technology (ICT) has transformed how people interact at work. Employees utilize the network to connect with their supervisors, coworkers, and subordinates as well as to fulfill their daily tasks. Organizations may use the network to coordinate and optimize employee work to promote efficiency (Yan and Zhong, 2018). According to reports, American users got around 97 legitimate business emails on average per day in 2019. Meanwhile, workers would send 30 business e-mails per day (Radicati Team, 2019). In China, 540 million individuals, accounting for 50.6% of all internet users, were using online applications for work as of December 2022 (China Internet Network Information Center, 2023). However, with the improvement of work and communication efficiency, cyber incivility is becoming more prominent.

Cyber incivility is a type of workplace behavior that acts against norms of mutual respect, and it emerges based on ICT (Lim and Teo, 2009; Giumetti et al., 2012). It is a common interpersonal stressor at work that may affect the daily lives of employees (Park et al., 2018). Over 90% of employees reported experiencing cyber incivility at work (Lim and Chin, 2006). Our pilot study of 1,499 current workers found that 75.22% of them had encountered cyber-incivility in China. Indeed, a growing amount of study (see review; Yan and Zhong, 2018) has linked it to health outcomes (e.g., negative emotions, physical distress, work burnout), behavioral outcomes (e.g., absenteeism, deviant workplace behaviors), and work outcomes (e.g., job performance, organizational commitment). Numerous studies have expanded on the findings in recent years. For instance, cyber incivility undermines basic need satisfaction, which has further detrimental impacts (Ju and Pak, 2021). It also serves as an essential mediator between race and perceived discrimination (Daniels and Thornton, 2020). However, the field of occupational health psychology currently still does not pay enough attention to cyber incivility. Cyber incivility is a two-dimensional structure. While passive cyber incivility has the “omission of respect” at its foundation, active cyber incivility has the “commission of disrespect” at its center (Yuan et al., 2020). Examples of active cyber incivility include saying unkind things about a victim in an email that they would never say in person or inserting caustic comments between paragraphs of an email. And examples of passive cyber incivility include when instigators ignore victims’ emails completely or react to victims but fail to address their concerns (Lim and Teo, 2009).

Despite the study that cyber incivility has been associated with negative well-being (Park et al., 2018), research on the impact of active versus passive cyber incivility on well-being (e.g., insomnia and negative emotions, Yuan et al., 2020; Zhou et al., 2022) suggests they might have distinct consequences. Therefore, further study is required to see whether they have differential effects on other well-being indicators, and we choose emotional exhaustion as the indicators, since it is seen as the typical result of being affected by high job demands, and it is also the result of the accumulation of negative emotions (Shin and Hur, 2022). Given that previous studies have explored the relationship between cyber incivility and negative emotions, this research pays specific attention to emotional exhaustion. Previous studies indicated a positive correlation between general cyber incivility and emotional exhaustion (Niven et al., 2022). As the active and passive dimensions may have different effects, we would like to analyze whether this difference exists on emotional exhaustion. This is the first important contribution we seek to make to the current incivility literature. By investigating the impact of the two-dimensional structure of cyber incivility on employee well-being, we aim to enrich studies of cyber incivility as well as make progress in the theory of workplace mistreatment.

Second, building on self-determination theory (SDT; Deci et al., 2017), we suggest that intrinsic motivation mediates the association between cyber incivility and employee well-being. Cyber incivility has multiple connotations; for instance, it might lead to a negative interpersonal experience since it goes against fundamental interpersonal standards in the workplace (Lim and Teo, 2009). Considering that the content of active cyber-incivility frequently entails the denial of employees’ competence and that passive cyber-incivility might passively cause work lag, it is also a negative work-related experience (Zhou et al., 2022). In general, cyber incivility is a big issue that might impair intrinsic motivation, resulting in a negative spillover impact on subsequent results. This study enhances our knowledge of how cyber incivility affects mental health by examining the potential mediation function of intrinsic motivation.

The third contribution is to enrich the knowledge of potential boundary condition for the negative influence of cyber incivility. According to Zhou et al. (2022), the discussion of the moderating mechanism of cyber incivility is far from enough. Even if several moderating variables have been explored in earlier studies, a comprehensive analysis of two-dimensional cyber incivility is lacking due to the use of cyber incivility composite scores or a focus on active cyber incivility only. SDT posits that intrinsic motivation is an internally adjusting continuum. Individual difference variables linked to goals, aspirations as well as work values are significant effect factors that can predict a variety of outcomes in different domains (Deci et al., 2017). This study attempts to investigate the moderating effect of promotion focus, which represents workers’ pursuit of goals and workplace performance. In the meantime, a comprehensive understanding of regulatory focus theory and self-determination theory will be discussed in conjunction with existing findings.

**Figure 1:**
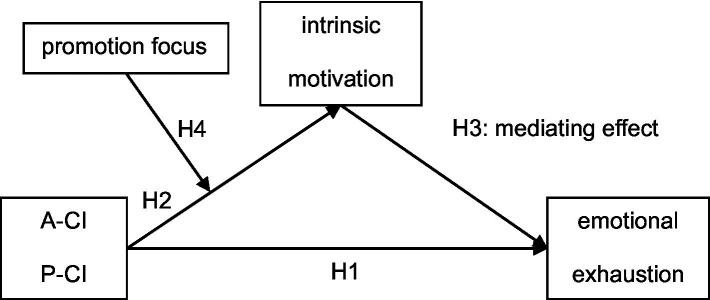
Conceptual model. A-CI, Active Cyber Incivility; P-CI, Passive Cyber Incivility.

In summary, the purpose of this study is to gain a better understanding of active and passive cyber incivility, as well as to analyze the relationship between emotional exhaustion and cyber incivility. Moreover, we hope to enrich the mediating mechanism literature on cyber incivility by drawing on self-determination theory and exploring the moderation of promotion focus, in order to expand and supplement the potential role of regulatory focus theory in cyber incivility. Figure 1 depicts the conceptual model.

## 2. Theoretical framework and hypothesis development

### 2.1. Self-determination theory

Self-determination theory is the primary theoretical foundation for this study. According to the general SDT model of work motivation, a workplace context that thwarts the satisfaction of employees’ basic psychological needs will affect their autonomous motivation. Specifically, individuals’ autonomous motivation will decline if they are unable to comprehend the value and purpose of their work, do not feel ownership and autonomy during the implementation process, or do not receive clear feedback and support. All of which will harm their reliability, learning, and adjustment (Deci et al., 2017). The principles of SDT help us comprehend how negative workplace events, such as cyber incivility, may affect employees’ well-being by altering their intrinsic motivation. Furthermore, given the self-regulating nature of intrinsic motivation, SDT provides a reference for the potential moderating function of individual difference characteristics in the linkage between cyber incivility and intrinsic motivation (Ryan and Deci, 2000). Our specific justifications and theories are presented in the parts that follow.

### 2.2. Cyber incivility and emotional exhaustion

As to Wirtz et al. (2017), emotional exhaustion is a condition of depletion and fatigue that happens when people feel they lack the resources to fulfill the duties that are expected of them (Chen et al., 2020). As shown by SDT, work situations that thwart need will be detrimental to employees’ health and wellness. We propose that the two dimensions of cyber incivility will positively predict emotional exhaustion. Active cyber incivility, in particular, which expresses instigators’ hostility through disrespectful statements (Yuan et al., 2020). Victims will experience greater emotional exhaustion because they will perceive that the mutual respect rule has been violated. Comparatively speaking, the intention of passive incivility is more ambiguous. Although the instigator does not perform rude behavior, he or she conveys disrespect by ignoring or delaying meeting the victim’s needs, which results in emotional exhaustion. As a result, we propose the following hypothesis:

*H1*: Both active (a) and passive (b) cyber incivility are positive predictors of emotional exhaustion.

### 2.3. Intrinsic motivation as a mediator

Intrinsic motivation is a specific type of autonomous motivation (Deci et al., 2017). It refers to a person’s innate propensity to seek out novelty or challenges, develop and use their skills, explore, and learn (Ryan and Deci, 2000). Previous studies have never focused on the connection between cyber incivility and intrinsic motivation. There is also no conclusive evidence from studies between face-to-face incivility and intrinsic motivation. A study based on data from 481 employees in China showed that supervisor incivility weakened employees’ intrinsic motivation (Liu et al., 2019). However, another study looked at how coworker and customer incivility affected the intrinsic motivation of service employees but found no significant predictive effect (Hur et al., 2016). Despite the fact that studies cannot give a clear direction of reference, we contend that cyber incivility reduces intrinsic motivation. SDT claims that social contexts can encourage or inhibit intrinsic motivation by supporting or hindering people’s internal psychological needs (Ryan and Deci, 2000). Studies have found that customer incivility impairs employee need satisfaction (Lin and Lai, 2019), and even exposure to (witnessing) workplace incivility degrades employees’ needs (Bureau et al., 2021). In addition, competency feedback that is positive boosts motivation in organizational communication, whereas feedback that implies incompetence diminishes it (Adams et al., 2017).

Meanwhile, the degree to which individuals are intrinsically motivated predicts persistence and performance in the workplace and wellness (Fishbach and Woolley, 2022). Intrinsic motivation reduces the extent to which employees experience emotional exhaustion (Ryan and Deci, 2000; Kammeyer-Mueller et al., 2016). Based on theoretical and empirical evidence, we propose the following hypothesis:

*H2*: Active (a) and passive (b) cyber incivility are negative predictors of intrinsic motivation.

*H3a*: Intrinsic motivation plays a mediating role between active cyber incivility and emotional exhaustion.

*H3b*: Intrinsic motivation plays a mediating role between passive cyber incivility and emotional exhaustion.

So far, we propose the above three hypotheses since active and passive cyber incivility comes from the same structure. We suggest that they will play roles in the same direction, but the influencing intensity may be different. Specifically, active cyber incivility seems more aggressive since it conveys hostility in a way that is rather straightforward. As a result, victims may perceive disrespect quickly. Passive cyber incivility is expressed ambiguously and may have less impact.

### 2.4. Promotion focus as an exacerbator of the effects of cyber incivility

Ryan and Deci (2000) proposed that intrinsic motivation is a process of internal regulation. Individual aspirations or values related to work are reliable individual difference variables that affect motivation (Deci et al., 2017). According to prior studies, regulatory focus theory is relevant to motivation and behavior, suggesting that people pursue their careers in two different ways (Brenninkmeijer et al., 2010). Employees who are focused on promotions adopt gain-maximizing tactics based on growth, aspiration, or accomplishment and are sensitive to the presence or absence of positive outcomes (Higgins and Pinelli, 2020). We infer that promotion focus exacerbates the negative effect of cyber incivility on intrinsic motivation. The reasons behind this are that, on the one hand, competence is one of the core cognitive components of intrinsic motivation. Employees who engage in promotion focus regard the goal of doing well at work as a kind of gain, and believe that good performance will allow them to reach their full professional and employment potential (Wallace and Chen, 2006). However, cyber incivility is a sign of disregard for employees’ abilities while undermining their desire to do their jobs well. On the other hand, promotion focus activates the employee’s pursuit of self-esteem, and in the case of failure, the self-esteem system will take a larger hit (Leonardelli et al., 2007). Cyber incivility is a threat to self-esteem. People seek to maintain a high feeling of self-worth in workplace communication, so they are more sensitive to hints of disrespect (Semmer et al., 2019). Employees with a high promotion focus may be more prone to perceive disrespectful treatment out of the need to maintain self-esteem, which has a more negative impact on intrinsic motivation. Taken together, we present the following hypothesis:

*H4a*: Promotion focus moderates the relationship between active cyber incivility and intrinsic motivation.

*H4b*: Promotion focus moderates the relationship between passive cyber incivility and intrinsic motivation.

Specially, the negative relationship between cyber incivility and intrinsic motivation is stronger for those with higher promotion focus than for those with lower promotion focus. Besides, promotion focus is involved with achievement, hence it may be more responsive to work-related factors. In view of the negative impact of passive cyber incivility on work progress (Zhou et al., 2022), we expect that compared with active cyber incivility, promotion focus plays a stronger moderating role for passive cyber incivility.

## 3. Overview of studies

We carried out two studies to examine our theoretical model (see Figure 1). In Study 1, we examined these effects using a scenario-simulation experiment to strengthen causal conclusions and exclude alternative hypotheses. In Study 2, we performed a two-wave time-lagged field study to retest the hypotheses and to examine the intertemporal effects of cyber incivility on emotional exhaustion.

### 3.1. Study 1

#### 3.1.1. Method

##### 3.1.1.1. Sample and procedure

A single-factor 3-level (condition: active vs. passive vs. control group) between-subjects design was employed. We calculated the participants’ number needed *via* G* power, and the effect size was set to be 0.25 indicating a medium effect, and 0.95 in power. It displayed that we should recruit at least 305 participants.

Altogether, 440 currently employed adults (63.2% female). They come from a variety of industries, including internet and e-commerce, education, healthcare, construction, manufacturing, and others, and are all full-time employees with an average age of 30.90 years (SD = 0.66) and an average working age of 7.27 years (SD = 0.99). The participants were randomly split into three groups of 151 (active group; 57.0% female), 148 (passive group; 66.2% female), and 141 (control group; 66.7% female), with an average age of 31.26 years (SD = 0.69) for the active group, 30.88 years (SD = 0.67) for the passive group, and 30.47 years (SD = 0.97) for the control group.

We used a scenario-simulation paradigm to manipulate the experimental conditions, and the participants were asked to conduct the experiment imaginatively in the simulated story situation. Scholars have used this paradigm in the manipulation of workplace incivility (Chui and Dietz, 2014). The importance of reading the scenario carefully was emphasized *via* online instructions. Having read the manipulation scenarios (see Appendix), participants completed a post-test questionnaire that included a manipulation check, related scales (including intrinsic motivation, emotional exhaustion and promotion focus), and demographic questions. Ten yuan were given to each participant.

##### 3.1.1.2. Manipulation of cyber incivility

To create these manipulations, real-life experiences of cyber incivility were collected. These experiences were combined with descriptions from the cyber incivility scale (Lim and Teo, 2009), and adapted to workplace incidents. Two episodes were developed for each experimental condition, for a total of six. We recruited 14 postgraduates (78.57% female, *M_age_* = 28.13) majoring in occupational health psychology to rate the degree of the incivility of each episode so as to validate the manipulations. They responded using a 5-point Likert scale with a range of 1 (no at all) to 5 (extreme uncivil). We collected suggestions about settings and descriptions to improve the episodes. Then we recruited 34 participants (82.35% female, *M_age_* = 28.65) to rate the episodes. Finally, based on the ratings, we chose the best appropriate episode for each level.

In active group, participants experience more offensive comments from supervisors. For example, supervisor always arranges work without telling the clear requirements, but he or she sends negative feedback after the participant completed work: “The work was not well considered at all. If you cannot do it, go home.” In passive group, participants experience more ambiguous communication with supervisor. For example, there is always no effective reply when participants ask about work issues, so that the work is perfunctory or delayed in various ways. In control group, the communication between participants and supervisor is always normal and effective (see Appendix for detail).

Episodes accord with the characteristics of active and passive cyber incivility (Yuan et al., 2020). According to Motro et al. (2021), gender affects people’s appraisal of incivility, so gender and other demographic information were not displayed in the manipulation scenario.

##### 3.1.1.3. Measures

Unless otherwise noted, items were assessed on a scale from (1) strongly disagree to (7) strongly agree.

###### 3.1.1.3.1. Manipulation check

Participants were asked to evaluate the degree of the incivility of the character’s behavior in the episode on a 5-point Likert scale where 1 = not at all to 5 = extremely.

###### 3.1.1.3.2. Intrinsic motivation

Participants were asked to indicate their agreement with a six-item measure of intrinsic motivation (Kuvaas, 2006; Cronbach’s α = 0.96). An example item was “The tasks that I do at work are enjoyable.” The Chinese version got from Yang (2016).

###### 3.1.1.3.3. Emotional exhaustion

Participants were asked to indicate their agreement with a five-item measure of emotional exhaustion (Li and Shi, 2003; Chinese version; Cronbach’s α = 0.95). An example item was, “Work makes me feel exhausted.”

###### 3.1.1.3.4. Promotion focus

Participants were asked to indicate their agreement with a nine-item measure of promotion focus (Neubert et al., 2008; Cronbach’s α = 0.80). An example item was “I take chances at work to maximize my goals for advancement.” The Chinese version came from Qi (2018).

###### 3.1.1.3.5. Control variables

Participants’ demographic characteristics (gender, age, educational background, tenure, position, industry) were controlled.

#### 3.1.2. Results

##### 3.1.2.1. Manipulation check

We performed a one-way 3-level (incivility conditions: active, passive, control) analysis of variance (ANOVA) with simple contrasts on our manipulation checks. There was a main effect on the evaluation of the degree of incivility, *F* (2, 439) = 479.37, *p* < 0.001, *η*^2^ = 0.67. The incivility rating of the control group (*M_control_* = 1.35, SD = 0.84) was significantly lower than that of the active group (*M_active_* = 4.40, SD = 0.87, contrast estimate = −3.05, SE = 0.11, *p* < 0.001) and the passive group (*M_passive_* = 4.15, SD = 1.06, contrast estimate = −2.79, SE = 0.11, *p* < 0.001). The incivility rating of the passive group was significantly lower than that of the active group, with contrast estimate = 0.26, SE = 0.11, *p* = 0.02. Therefore, our manipulations were effective.

##### 3.1.2.2. Hypothesis testing

To test Hypotheses, we first examined whether our manipulations influenced intrinsic motivation and emotional exhaustion, then we used the bootstrapping approach (Hayes, 2018) to test the mediating effect of intrinsic motivation and the moderating effect of promotion focus [referred to Motro et al. (2021) and Vincent and Kouchaki (2016)].

###### 3.1.2.2.1. Emotional exhaustion

A one-way ANOVA with incivility conditions on emotional exhaustion was conducted. Results demonstrated that incivility conditions had a significant effect, *F*(2, 439) =115.35, *p* < 0.001, *η*^2^ = 0.35. What we found from the pairwise comparisons were that emotional exhaustion in control condition (*M_control_* = 3.02, SD = 1.45) was significantly lower than it in active condition (*M_active_* = 5.41, SD = 1.29), contrast estimate = −2.39, SE = 0.17, *p* < 0.001, 95% CI [−2.72, −2.06]; and it was also lower than in passive condition (*M_passive_* = 4.99, SD = 1.53), contrast estimate = −1.96, SE = 0.17, *p* < 0.001, 95% CI [−2.29, −1.63]. Moreover, emotional exhaustion in active condition was significantly higher than it in passive condition, contrast estimate = 0.43, SE = 0.16, *p* = 0.01, 95% CI [0.10, 0.75]. Hypothesis 1 was supported.

###### 3.1.2.2.2. Intrinsic motivation

We ran a one-way ANOVA with incivility conditions on intrinsic motivation. We found that incivility conditions had a significant effect, *F*(2, 439) =147.90, *p* < 0.001, *η*^2^ = 0.40. Results of pairwise comparisons showed that intrinsic motivation in control condition (*M_control_* = 5.63, SD = 0.81) was significantly higher than it in active condition (*M_active_* = 3.06, SD = 1.51), contrast estimate = 2.57, SE = 0.16, *p* < 0.001, 95% CI [2.26, 2.89]; and it was also higher than in passive condition (*M_passive_* = 3.39, SD = 1.65), contrast estimate = 2.24, SE = 0.16, *p* < 0.001, 95% CI [1.92, 2.56]. Besides, intrinsic motivation in passive condition was significantly higher than it in active condition, contrast estimate = 0.33, SE = 0.16, *p* = 0.04, 95% CI [0.02, 0.65]. Hypothesis 2 was supported.

###### 3.1.2.2.3. Mediation

Table 1 displays the correlations among the study variables.^1^

**Table 1:** Descriptive statistics and correlations among Study 1 variables.

Variable	*M*	SD	1	2	3	4	5
1. A	0.34	0.48	—				
2. P	0.34	0.47	−0.51^***^	—			
3. C	0.32	0.47	−0.49^***^	−0.50^***^	—		
4. intrinsic motivation	4.00	1.78	−0.39^***^	−0.25^***^	0.65^***^	—	
5. emotional exhaustion	4.50	1.76	0.38^***^	0.20^***^	−0.58^***^	−0.74^***^	—
6. promotion focus	5.29	0.75	−0.02	0.05	−0.03	0.02	0.03

*N* = 440. Table shows the results of partial correlation analysis. A was coded as 1 = active cyber incivility, 0 = all other groups. P was coded as 1 = passive cyber incivility, 0 = all other groups. C was coded as 1 = control group, 0 = all other groups. Two-tailed: ^***^p < 0.001.

To test the mediating effect of intrinsic motivation (Hypotheses 3), we ran the macro PROCESS (v4.0, Model 4) in SPSS 26.0 by Hayes (2018). During the process, two dummy variables were created with conditions as the focal comparison group. Specifically, X1 was coded as 1 = active cyber incivility and 0 = all other groups. X2 was coded as 1 = passive cyber incivility and 0 = all other groups. The control condition was represented by X1 = 0 and X2 = 0 and thus served as the reference group. The X1 code represented a comparison between the active and control group, whereas X2 represented a comparison between the passive and control group. We entered emotional exhaustion as the dependent variable; and performed bootstrapping on 5,000 samples.

Table 2 displays all regression coefficients.

**Table 2:** Regression coefficient estimates in Study 1 with control group as referent group.

Variables	Intrinsic motivation			Emotional exhaustion		
	*B*	Boot *SE*	Boot 95% CI	*B*	Boot *SE*	Boot 95% CI
Constant	5.91^***^	0.47	[5.00, 6.82]	7.06^***^	0.57	[5.97, 8.18]
X1	−2.62^***^	0.13	[−2.87, −2.36]	0.80^***^	0.20	[0.39, 1.19]
X2	−2.25^***^	0.15	[−2.53, −1.95]	0.58^**^	0.21	[0.15, 0.98]
IM				−0.62^***^	0.05	[−0.72, −0.52]
PF	0.35^*^	0.09	[0.18, 0.52]			
X1 × PF	−0.28	0.18	[−0.69, 0.01]			
X2 × PF	−0.51^*^	0.23	[−1.02, −0.13]			
gender	0.03	0.03	[−0.24, 0.29]	0.17	0.12	[−0.05, 0.39]
age	0.04	0.14	[−0.25, 0.31]	−0.25	0.14	[−0.53, 0.02]
education	−0.33^**^	0.11	[−0.54, −0.10]	−0.19	0.12	[−0.42, 0.05]
tenure	−0.10	0.09	[−0.27, 0.09]	0.09	0.08	[−0.07, 0.25]
position	0.58^***^	0.10	[0.39, 0.78]	0.11	0.10	[−0.08, 0.31]
industry	−0.03	0.04	[−0.10, 0.05]	−0.05	0.03	[−0.11, 0.01]
*R^2^*	0.48			0.57		
*F*	36.40^***^			64.46^***^		

*N* = 440. X1 was coded as 1 = active cyber incivility, 0 = all other groups. X2 was coded as 1 = passive cyber incivility, 0 = all other groups. The control group was represented by X1 = 0 and X2 = 0, and thus served as the reference group. IM, intrinsic motivation; PF, promotion focus. ^*^p < 0.05, ^**^p < 0.01, ^***^p < 0.001.

Results demonstrated that the total effect of incivility conditions on emotional exhaustion was significant, *B_X1_* = 2.41, SE = 0.17, *p* < 0.001, 95% CI [2.08, 2.74]; *B_X2_* = 1.97, SE = 0.17, *p* < 0.001, 95% CI [1.64, 2.29]. Then both X1 (*B* = 0.80, SE = 0.17, *p* < 0.001, 95% CI [0.45, 1.15]) and X2 (*B* = 0.58, SE = 0.17, *p* = 0.001, 95% CI [0.25, 0.90]) had significant direct effects on emotional exhaustion. Hypothesis 1 was supported again.

Next, X1 significantly negatively predicted intrinsic motivation, *B* = −2.62, SE = 0.16, *p* < 0.001, 95% CI [−2.92, −2.31]; so did X2, *B* = −2.25, SE = 0.15, *p* < 0.001, 95% CI [−2.55, −1.95]. Hypothesis 2 was supported additionally.

Intrinsic motivation demonstrated a strong effect on emotional exhaustion, *B* = −0.62, SE = 0.04, *p* < 0.001, 95% CI [−0.70, −0.54]. The indirect effects of incivility conditions on emotional exhaustion through intrinsic motivation were significant, X1: *indirect effect* = 1.62, *BootSE* = 0.15, *BootLLCI* = 1.35, *BootULCL* = 1.93, and X2: *indirect effect* = 1.39, *BootSE* = 0.15, *BootLLCI* = 1.12, *BootULCL* = 1.70; Zero was excluded in both confidence intervals, so Hypothesis 3 was supported.

###### 3.1.2.2.4. Moderation

To examine the moderating effect of promotion focus, we ran macro PROCESS (v4.0, Model 7) in SPSS 26.0 by Hayes (2018). The creation of dummy variables, bootstrapping setting and other processes were same as the mediation’s approach.

**Figure 2:**
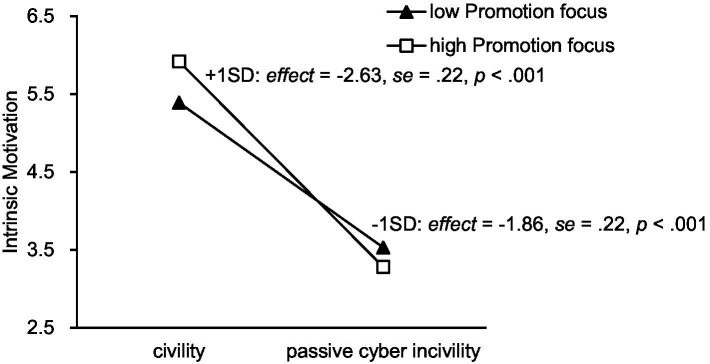
Interaction effect of incivility condition and promotion focus on intrinsic motivation.

We found that the effect of the interaction between X2 and promotion focus was significant, *B* = −0.51, SE = 0.21, *p* = 0.01, 95% CI [−0.92, −0.11], and zero was not included in the confidence interval; but the effect of the interaction between X1 and promotion focus was not significant, *B* = −0.28, SE = 0.20, *p* = 0.17, 95% CI [−0.67, 0.12], and zero was included in the confidence interval. Only Hypothesis 4b was supported. Simple slope analyses (see Figure 2) revealed that the negative effect of passive cyber incivility on intrinsic motivation was more pronounced in the group with a higher promotion focus (see Table 2 for all regression coefficients).

**Figure 3:**
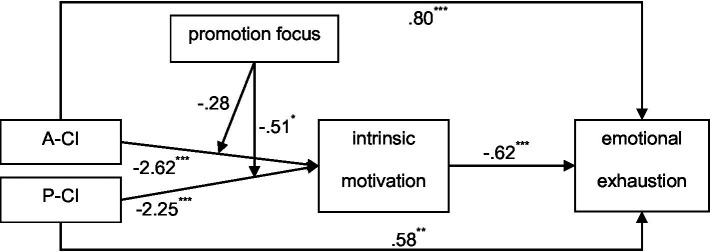
Overall results of Study 1. A-CI, Active cyber incivility; P-CI, Passive cyber incivility. ^*^p < 0.05, ^**^p < 0.01, ^***^p < 0.001.

We present the results in the conceptual diagram (see Figure 3).

###### 3.1.2.2.5. Supplementary analysis^2^

We conducted supplementary analyses to examine whether there was a significant intensity difference between active and passive cyber incivility of the mediation. Active condition was coded as 1, passive condition was coded as 0, and control condition was excluded; so passive condition served as the reference group.

Results showed that the indirect effect of intrinsic motivation was 0.21, *BootSE* = 0.10, *BootLLCI* = 0.01, *BootULCL* = 0.40.

#### 3.1.3. Discussion

The findings from Study 1 provide empirical and theoretical support for the notion that intrinsic motivation is an important mediating mechanism of cyber incivility. Strong evidence for the empirical and theoretical distinctions between active and passive cyber incivility is also supported. Promotion focus is found to moderate the effect of passive cyber incivility on intrinsic motivation but not active cyber incivility. Results also show that passive cyber incivility has a weaker influence than active cyber incivility. Therefore, our results confirm that active and passive cyber incivility should not be represented by cyber incivility composite scores, which will cover up their theoretical richness.

### 3.2. Study 2

#### 3.2.1. Method

##### 3.2.1.1. Sample and procedure

To take part in a study on workplace experiences, we enlisted 300 full-time workers from Credamo (Study 1 participants were not included in this recruitment). Of the 300 recruits, 235 (68.9% female; *M_age_* = 30.85, SD = 0.67; *M_tenure_* = 8.03, SD = 1.07) completed both questionnaires for a response rate of 78.33%. No significant demographic differences were found between respondents and nonrespondents, suggesting no systematic differences between the two groups. All participants were informed that participation was voluntary and their responses would be kept confidential to reduce potential social desirability bias. After providing informed consent, they completed two questionnaires one week apart. The first survey collected data on cyber incivility, intrinsic motivation, promotion focus, and demographics; the second survey provided data on emotional exhaustion [referred to Welbourne and Sariol (2017)]. After completing the first and second questionnaires, the participants were paid 5 yuan, respectively.

##### 3.2.1.2. Measures

Unless otherwise noted, items were assessed on a scale from (1) strongly disagree to (7) strongly agree.

###### 3.2.1.2.1. Cyber incivility

We used Lim and Teo’s (2009) publicly published scale and conducted a standard scale revision based on 1,499 Chinese currently employed persons (54.97% female; *M_age_* = 32.95, SD = 0.78; *M_tenure_* = 8.85, SD = 1.07) before the formal test. In the Chinese version of the cyber incivility scale, the active dimension retained items 1–6, and the passive dimension retained items 1, 3, 4, and 7.^3^ An example of an active item was “Said something hurtful to you through online communication.” An example passive item was “Replied to your messages but did not answer your queries.” Participants were asked to indicate their agreement with a ten-item measure of cyber incivility, and items were assessed on a scale from (1) never to (5) always happens. In this study, the total scale’s Cronbach’s α = 0.91, the active subscale’s Cronbach’s α = 0.90, and the passive subscale’s Cronbach’s α = 0.82.

###### 3.2.1.2.2. Intrinsic motivation

Same as study 1; Cronbach’s α = 0.94.

###### 3.2.1.2.3. Emotional exhaustion

Same as study 1; Cronbach’s α = 0.94.

###### 3.2.1.2.4. Promotion focus

Same as study 1; Cronbach’s α = 0.85.

###### 3.2.1.2.5. Control variables

Same as study 1.

#### 3.2.2. Results

##### 3.2.2.1. Common method bias check

Harman’s one-factor test and CFA were used to explore whether our results were susceptible to common method bias. Common method variance may be not significant if an exploratory factor analysis (EFA) produces one factor that accounts for less than 40% of the covariance among the variables (Tang and Wen, 2020). In our case, five factors, each with an eigenvalue larger than 1.0, emerged from the unrotated EFA, and the largest of the five retained factors accounted for 32.62%. We then conducted CFA to examine the factorial validity of the five measures (active cyber incivility, passive cyber incivility, intrinsic motivation, emotional exhaustion, and promotion focus). The results showed that the five-factor model fit our data well, χ^2^(398) = 732.77, *p* < 0.001, CFI = 0.93, TLI = 0.93, RMSEA = 0.06, and was significantly better than a four-factor model where all cyber incivility items were combined with Δχ^2^(3) = 134.30, *p* < 0.001, a three-factor model where intrinsic motivation and promotion focus items were combined with Δχ^2^(4) = 565.09, *p* < 0.001, a two-factor model where cyber incivility and emotional exhaustion items were combined with Δχ^2^(5) = 1791.53, *p* < 0.001, or a one-factor with Δχ^2^(7) = 2173.95, *p* < 0.001, demonstrating that all factor loadings were statistically significant with a good model fit and provided evidence for the distinction among constructs.

##### 3.2.2.2. Hypothesis testing

Table 3 displays the correlations among the study variables.^4^

**Table 3:** Descriptive statistics and correlations among Study 2 variables.

Variable	*M*	SD	1	2	3	4
1. active cyber incivility	1.86	0.78	—			
2. passive cyber incivility	2.46	0.88	0.78^***^	—		
3. intrinsic motivation	4.65	1.44	−0.26^***^	−0.41^***^	—	
4. emotional exhaustion	3.80	1.60	0.37^***^	0.44^***^	−0.52^***^	—
5. promotion focus	4.97	0.86	0.09	0.04	0.41^***^	−0.31^***^

*N* = 235. Table shows the results of partial correlation analysis. Two-tailed: ^***^p < 0.001.

We put active and passive cyber incivility into independent variables, respectively. We set to mean center of the continuous variables to reduce multicollinearity (Luo and Jiang, 2012) and performed bootstrapping of 5,000 samples. Then we ran Model 4 to examine the total effect, direct effect and mediating effect. Afterward, we ran Model 7 to test the moderating effect. All regression analyses used the macro PROCESS (v4.0) in SPSS 26.0.

Tables 4, 5 display all regression coefficients.

**Table 4:** Regression coefficient estimates of active cyber incivility in Study 2.

Variables	Intrinsic motivation			Emotional exhaustion		
	*B*	Boot *SE*	Boot 95% CI	*B*	Boot *SE*	Boot 95% CI
Constant	3.49^***^	0.66	[2.18, 4.79]	6.06^***^	0.71	[4.72, 7.51]
A-CI	−0.58^***^	0.11	[−0.79, −0.37]	0.47^***^	0.13	[0.19, 0.70]
IM				−0.51^***^	0.08	[−0.67, −0.37]
PF	0.70^***^	0.09	[0.52, 0.87]			
A-CI × PF	0.12	0.13	[−0.19, 0.34]			
gender	0.17	0.18	[−0.18, 0.51]	0.12	0.18	[−0.24, 0.47]
age	0.38^*^	0.18	[0.03, 0.73]	−0.22	0.22	[−0.64, 0.20]
education	−0.07	0.13	[−0.32, 0.19]	0.09	0.17	[−0.24, 0.41]
tenure	0.05	0.13	[−0.20, 0.30]	−0.05	0.14	[−0.33, 0.23]
position	0.14	0.13	[−0.13, 0.40]	0.13	0.15	[−0.15, 0.42]
industry	−0.01	0.05	[−0.12, 0.08]	0.02	0.05	[−0.08, 0.11]
*R^2^*	0.33			0.37		
*F*	12.07^***^			16.39^***^		

*N* = 235. A-CI, active cyber incivility; IM, intrinsic motivation; PF, promotion focus. ^*^p < 0.05, ^***^p < 0.001.

**Table 5:** Regression coefficient estimates of passive cyber incivility in Study 2.

Variables	Intrinsic motivation			Emotional exhaustion		
	*B*	Boot *SE*	Boot 95% CI	*B*	Boot *SE*	Boot 95% CI
Constant	3.60^***^	0.47	[−1.68, 0.15]	5.96^***^	0.48	[−0.95, 0.89]
P-CI	−0.61^***^	0.06	[−0.50, −0.26]	0.43^***^	0.06	[0.12, 0.37]
IM				−0.49^***^	0.07	[−0.57, −0.30]
PF	0.70^***^	0.05	[0.31, 0.53]			
P-CI × PF	0.01	0.05	[−0.11, 0.10]			
gender	0.15	0.12	[−0.12, 0.33]	0.11	0.11	[−0.15, 0.28]
age	0.37^*^	0.12	[0.01, 0.51]	−0.22	0.13	[−0.40, 0.12]
education	−0.03	0.09	[−0.19, 0.16]	0.06	0.11	[−0.19, 0.25]
tenure	0.00	0.09	[−0.18, 0.18]	−0.03	0.09	[−0.19, 0.15]
position	0.16	0.09	[−0.06, 0.28]	0.10	0.09	[−0.11, 0.25]
industry	−0.02	0.03	[−0.08, 0.05]	0.03	0.03	[−0.05, 0.08]
*R^2^*	0.38			0.37		
*F*	15.07^***^			16.39^***^		

*N* = 235. P-CI, passive cyber incivility; IM, intrinsic motivation; PF, promotion focus. ^*^p < 0.05, ^**^p < 0.01, ^***^p < 0.001.

The results showed that the total effects of active and passive cyber incivility on emotional exhaustion were significant, *B_active_* = 0.71, SE = 0.12, *p* < 0.001, 95% CI [0.48, 0.95]; *B_passive_* = 0.71, SE = 0.11, *p* < 0.001, 95% CI [0.50, 0.92].

Active cyber incivility directly predicted emotional exhaustion, *B* = 0.47, SE = 0.11, *p* < 0.001, 95% CI [0.25, 0.69]; as well as passive cyber incivility, *B* = 0.43, SE = 0.10, *p* < 0.001, 95% CI [0.23, 0.63]. Hypothesis 1 was supported.

Active and passive cyber incivility had significant negative relationships with intrinsic motivation, *B_active_* = −0.58, SE = 0.10, *p* < 0.001, 95% CI [−0.78, −0.37]; *B_passive_* = −0.61, SE = 0.09, *p* < 0.001, 95% CI [−0.78, −0.41]. Hypothesis 2 was supported.

The indirect effect of intrinsic motivation between active cyber incivility and emotional exhaustion was 0.30, *BootSE* = 0.07, *BootLLCI* = 0.17, *BootULCL* = 0.45; and the indirect effect in passive dimension was 0.30, *BootSE* = 0.06, *BootLLCI* = 0.19, *BootULCL* = 0.43. There were no zeros including in both confidence intervals, and Hypothesis 3 was supported.

Last, the results demonstrated that promotion focus did not moderate the relationship between cyber incivility and intrinsic motivation. The effect of the interaction between active cyber incivility and promotion focus on intrinsic motivation was 0.12, SE = 0.11, *p* = 0.29, 95% CI [−0.10, 0.33]; neither did passive cyber incivility, *B* = 0.01, SE = 0.09, *p* = 0.94, 95% CI [−0.17, 0.18]. Hypothesis 4 was not supported.

**Figure 4:**
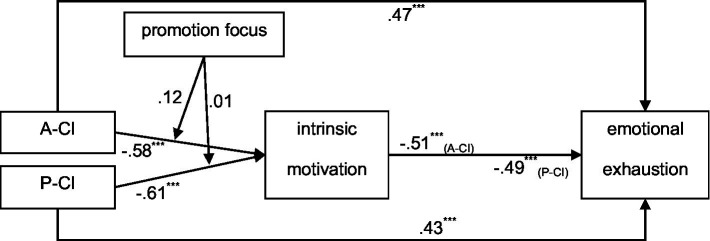
Overall results of Study 2. A-CI, Active Cyber Incivility; P-CI, Passive Cyber Incivility. ^***^p < 0.001.

The overall results were presented in the conceptual diagram; see Figure 4.

#### 3.2.3. Discussion

Study 2 revealed evidence in favor of the negative effects of both active and passive cyber incivility, which is consistent with our reasoning. Unlike Study 1’s findings, the moderating effect of promotion focus is not significant.

## 4. General discussion

We have conducted two studies to look further into cyber incivility in the workplace. By paying attention to its two-dimensional structure and their potential differences, the present research highlights the value of a nuanced study of cyber incivility. Both experimental and time-lagged studies have found that cyber incivility is a positive predictor to emotional exhaustion, and intrinsic motivation is the critical mediating mechanism. However, there is no consistent conclusion on the moderating role of promotion focus.

### 4.1. Theoretical and research implications

This research makes a few contributions to the literature. First, the literature on the causal relationship between cyber incivility and employee well-being has been enriched. The indicator of well-being explored in this research is emotional exhaustion, which predicts subsequent health outcomes (Bakker and Demerouti, 2017). The present research has found that the direct negative effect of cyber incivility on emotional exhaustion has an intertemporal effect (lasting at least one week). This finding reminds us that it may have a prolonged adverse effect on employees’ health even if cyber incivility is a mild form of workplace abuse.

Our research also supports the need to pay more attention to the two-dimensional structure of cyber incivility and provides a clearer understanding of the influences of active and passive cyber incivility. Active cyber incivility has a stronger impact (on intrinsic motivation and emotional exhaustion) than passive incivility in the scenario experiment. This suggests that the effect of active cyber incivility is stronger during exposure to a rude situation because hostility is expressed more obviously (Yuan et al., 2020) and thus the victim clearly feels offended. The manipulation check also provides evidence that the incivility score of the passive situation is significantly lower than that of the active situation, indicating that it conveys less direct hostility. However, the results of the time-lagged study appear to be different: the strength of the effect of active and passive dimensions seems to be similar. This is likely because the intent of passive cyber incivility is so vague that it takes time for the victim to clarify the instigator’s intention, at the same time, the victim may have engaged in more rumination (Zhou et al., 2022), making the negative influence of the passive dimension increase gradually. Combined with the conclusions of previous studies (Yuan et al., 2020; Zhou et al., 2022), we believe that the results of this research reflect the potential difference in the influences of active and passive cyber incivility; that is, both of them have negative impacts on employee well-being. But the influence of active cyber incivility tends to be immediate and gradually weakens over time, whereas the impact of passive cyber incivility is a slow process that may last longer and gradually increase in intensity. In sum, our research supports the importance of distinguishing between the commission of disrespect and the omission of respect in the field of work abuse.

Second, our research expands the literature on the underlying mechanisms of cyber incivility by demonstrating that both active and passive cyber incivility can destruct intrinsic motivation. A decrease in intrinsic motivation indicates that employees’ psychological needs are frustrated (Fishbach and Woolley, 2022); the satisfaction of psychological needs will make employees enjoy work more and reduce burnout (Deci et al., 2017). The findings provide a new reference for the underlying mechanism between cyber incivility and negative emotions explored in previous studies given that emotional exhaustion is the result of the accumulation of various negative emotions (Spagnoli and Molinaro, 2020). In addition, intrinsic motivation is a precious resource in itself. When work is mostly intrinsically motivated, people feel that they are integrated with the task, which produces positive psychological experiences (Fishbach and Woolley, 2022). This research indicates that cyber incivility destroys employees’ internal love for work, and they cannot mobilize their own resources to deal with negative job events, resulting in an increase in the depletion of emotional resources.

Third, although the moderating effect of promotion focus has not been completely proven, it also contributes to understanding the boundary condition of cyber incivility. According to the findings of Study 1, employees with high promotion focus are more likely to experience a decrease in intrinsic motivation after experiencing to passive cyber incivility than those with low promotion focus. Study 2 does not capture the moderating effect of promotion focus, most likely as a result of the different research methods. The outcomes of the scenario-simulation experiment depict how workers might react immediately to a harsh situation, while the results of the multi-wave questionnaire show how things generally go at work. Promotion focus improves people’s subjective initiative (Auh et al., 2022) and promotes employees to proactively change at work to improve intrinsic motivation (Smith et al., 2009). Thus, the active adjustment may lessen the sensation of collapse brought on by cyber-incivility. Compared with the negative state of the immediate reaction, the negative outcome of cyber incivility, despite not significantly improving, is at least not exacerbated. As a result, the moderating effect of promotion focus disappears.

### 4.2. Practical implications

Our findings also provide important implications for workplace practice. First, we demonstrate the negative impact of different manifestations of cyber incivility on employee well-being. Organizations should be aware of the detrimental impacts of cyber incivility and the importance of preventing or stopping it without delay. Formulating civilized cyber communication norms is a necessary beginning. Under clear communication norms, the ambiguous boundaries of cyber incivility are clarified to a great extent, and the frequency of inadvertently inciting rude behavior may be reduced when employees have a clear understanding of unacceptable or undesirable behavior. To improve employees’ self-regulation and reduce the loss of well-being caused by cyber incivility, organizations should establish relevant policies. For example, companies could add stress-management training program, or set up HR hotlines (Hur et al., 2016).

Second, our research shows that cyber incivility diminishes intrinsic motivation, indicating that employees’ autonomous states are affected. In a state of high intrinsic motivation, employees seek to complete tasks autonomously rather than doing things for external goals such as status or money (Fishbach and Woolley, 2022). Organizations is supposed to encourage employees to be motivated by their inner happiness. At the same time, standardized work processes should be established to give employees more autonomy. For instance, what measures can be taken by employees in cases where they do not receive an effective response for a long time. The organization’s workflow ought to include applicable coping measures and policies. Instead of making employees responsible for all negative outcomes, the organization should define the parameters of employees’ independent decision-making, how to report when those criteria are exceeded, and how to divide responsibilities when a report is submitted on time but is not addressed promptly. For active cyber incivility, feedback that implies employees’ incompetence threatens intrinsic motivation (Adams et al., 2017), so it is best to start communicating by affirming the advantages of employees and then proposing directions for improvement. Win-win results are produced for both employees and the organization when communication is conducted in an appropriate way that meets the psychological needs of employees from either a short- or long-term view.

Last but not least, our research demonstrates that promotion focus may exacerbate the negative effects of passive cyber incivility. We propose that regardless of whether employees strongly pursue positive outcomes or are highly alert to negative outcomes, it will worsen the effect of negative work events. This is in line with the finding of a previous study (Zhou et al., 2022), which indicates that prevention focus aggravates the influence of active cyber incivility. Organizations may help employees to find a balance between success and failure, thus avoiding excessive stress. Moreover, the advantages and disadvantages of promotion and prevention focus rely on how well individuals’ strategies fit with their surroundings (including the jobs they must complete) or how well regulations match. This level of fitness may have an impact on work performance, changes in attitude and behavior, and judgment and decision-making (Higgins, 2005; Higgins and Pinelli, 2020). In order to enhance the regulatory fit between people and the work environment and to increase employee well-being, employers might create customized interventions.

### 4.3. Limitations and directions for future research

There are four limitations that are worth discussing in the current research. First, although we use different methods to explore the relationship among variables, the hidden drawbacks of the self-reporting method cannot be avoided because all data were reported by subjects. Future research may adopt methods like third-party reporting of dependent variables to reduce the bias of subjects’ self-reports or use experiments to change mediating and moderating factors (Vincent and Kouchaki, 2016) to make the data and causal relationships between variables even more objective and clear.

The second one is about the manipulation of cyber incivility. On the one hand, the context of cyber incivility set by our scenario-simulation experiment occurred mainly in private conversations, except one subplot involving group chats. Actually, much work communication takes place in group chat, even a lot of cyber incivility occurs in social media with a wider audience (e.g., Facebook; Wolter et al., 2023). Thus, in addition to the instigators and victims, the bystanders of cyber incivility are important perspectives. Kim (2022) suggests that when incivility becomes the “new normal” in online communication, emphasis should be placed on the influence and process of bystanders, who probably act as interveners or reinforcers. Referring to the studies of workplace incivility (e.g., Miner and Cortina, 2016; Adiyaman and Meier, 2022) from the third-party perspective, the indirect impact of negative work-related events on bystanders also reflects their ripple effect. Therefore, future research can take bystanders into account to examine the impact of the third-party perspective on the interaction process of cyber incivility, or the effect of cyber incivility on them, so as to form a more three-dimensional understanding. On the other hand, we have manipulated cyber incivility from two dimensions, positive and negative. In order to increase nuanced understanding, future research can add other dimensions (e.g., degree of rudeness, accidentally vs. intentionally) to distinguish cyber incivility in more detail or discover more potential boundary conditions.

Thirdly, we find that the mechanisms of active and passive cyber incivility are similar. Even though we have talked about how the two are different in terms of intensity, it’s hard to discern more differences in other ways. Zhou et al. (2022) used interpersonal and work-related factors to tell the difference between active and passive dimensions, which is a good reminder. Future researchers can try to look into other moderators combined with characteristics of the two dimensions, which will give us a better and deeper understanding.

Moreover, the study only focused on the Chinese workplace context. In the future, cross-cultural studies can be attempted to enrich the potential influence of cultural background factors on cyber incivility. Due to the ambiguity of cyber incivility (Lim and Teo, 2009), individuals are more likely to be affected by the atmosphere or context when they perceive or evaluate it. Cultural context is pivotal in shaping personal cognition because it’s a common normative system guiding belief, emotion, and behavior (Chen et al., 2019). Cross-cultural research may thus be useful in clarifying the boundaries of cyber incivility and other important content.

## 5. Conclusion

The research endeavors to provide more insight into whether and how two-dimensional cyber incivility impacts employee well-being. Research results indicate that the mechanisms of the two-dimension of cyber incivility are similar. The detrimental impacts of active and passive cyber incivility on employees’ emotional exhaustion have been proven, and intrinsic motivation is a critical mediator. However, which of the two dimensions will be more effective in their roles? We are of the opinion that there is not only one answer. Referring to prior studies, time factor should be considered to answer the question, which also reflects the potential differences in their mechanisms. Overall, this research has reaffirmed the importance of cyber incivility based on the previous research. It also underlines that the research of work abuse can be concerned with the consequences, mechanisms, and differences in various presentations of disrespect. We conclude with a call for scholars to take a broader perspective of cyber incivility through more detailed research.

## Data availability statement

The raw data supporting the conclusions of this article will be made available by the authors, without undue reservation.

## Ethics statement

The studies involving human participants were reviewed and approved by the ethics committee of School of Philosophy of Wuhan University. The patients/participants provided their written informed consent to participate in this study.

## Author contributions

S-PX, YL, YY, ZZ, Z-XC, and K-CZ: contributed to the study conception and design. S-PX and YL: conceptualization. S-PX and YL: methodology. S-PX, YL, Z-XC, and K-CZ: material preparation. S-PX: formal analysis and investigation. S-PX and YL: writing—original draft preparation. S-PX, YL, YY, and ZZ: writing—review and editing. All authors read and approved the final manuscript.

## Conflict of interest

The authors declare that the research was conducted in the absence of any commercial or financial relationships that could be construed as a potential conflict of interest.

## Publisher’s note

All claims expressed in this article are solely those of the authors and do not necessarily represent those of their affiliated organizations, or those of the publisher, the editors and the reviewers. Any product that may be evaluated in this article, or claim that may be made by its manufacturer, is not guaranteed or endorsed by the publisher.

## Footnotes

^1^Considering the high correlation coefficients between intrinsic motivation and emotional exhaustion (−0.74), we conducted HTMT analysis to examine discriminant validity, and the index was 0.77, less than 0.90, indicating that the variables had good discriminant validity (Henseler et al., 2015). Next, we conducted confirmatory factor analysis (CFA) to test the factorial validity of three measures (intrinsic motivation, emotional exhaustion and promotion focus). We found the three-factor model fit the data well, x2 (165) = 440.52, *p* < 0.001, comparative fit index (CFI) = 0.96, Tucker-Lewis index (TLI) = 0.95, root mean square error of approximation (RMSEA) = 0.06, and was significantly better than a two-factor model where intrinsic motivation and promotion focus items were combined with Δx2(2) = 585.73, *p* < 0.001, a one-factor model where all items were combined with Δx2(5) = 1864.50, *p* < 0.001. It provided evidence for the distinction among constructs.

^2^In fact, the results of ANOVA had shown some differences in the effects of active and passive cyber incivility on intrinsic motivation and emotional exhaustion, and considering the moderating effect of promotion focus was not significant in the active cyber incivility condition, so we examined the potential difference of mediation.

^3^Please contact the authors to view the revised data of the scale.

^4^We used Amos 24.0 to calculate the discriminant validity between active and passive cyber incivility given that their correlation was high (0.78). HTMT analysis demonstrated that the index was 0.81, less than 0.90, indicating a good discriminant validity (Henseler et al., 2015).
