# Supplementary material for: Effects of two-dimensional cyber incivility on employee well-being from a self-determination perspective
Source: Front Psychol. 2023 Apr 11;14:1137587. doi: 10.3389/fpsyg.2023.1137587 (PMC10126297; doi:10.3389/fpsyg.2023.1137587)
Supplement: Supplementary file 1 [file Presentation_1.pdf]

Appendix: Experimental materials in Study 1

English Version

Active Cyber Incivility Condition

You’re an ordinary employee, and your daily work is mainly communicated and coordinated via online apps. Recently, you have been communicating with your supervisor as follows. He or she always did not state the requirements clearly when arranging work, whereas they just asked you to do it. However, he sent the feedback after you finished the work: " The work was not well considered at all. If you cannot do it, go home." After that, you reported the reasons for the stagnation and disadvantages when the project encountered problems, in order to adjust the plan. But the leader responded to you: " I don't know what you can do, use your head, okay?" Because of the disagreement, you had an argument with the leader. In the group chat, the leader insinuated that you didn't obey the management and sent messages in a strange tone: “Someone is really wasting your talent by staying in the company”.

Passive Cyber Incivility Condition

You’re an ordinary employee, and your daily work is mainly communicated and coordinated via online apps. Recently, you have been communicating with your supervisor as follows. When you ask for work instructions, there is always no effective reply, and the work is perfunctory or delayed in various ways. Once, the client encountered problems that you couldn’t handle, so you sent a message to the leader for help. The leader only replied "Oh" and did not answer the question. You asked the subsequent questions immediately but did not get any reply, and you had to apologize to the client. Yesterday, you submitted a document and sent it to the leader for review. The app showed that the leader had read the message, but you didn’t get any reply or get through the phone. At the same time, the leader forwarded the company's advertisement in the group chat.

Control Condition

You’re an ordinary employee, and your daily work is mainly communicated and coordinated via online apps. Recently, you have been communicating with your supervisor as follows. The leader always sends clear requirements when arranging work and answers your questions promptly when there is confusion. There were problems in the progress of the project last week. You reported the reasons for the stagnation and the disadvantages of the project and put forward to adjust the plan. The leader replied to you: “Well done.” You react promptly. “Please make a plan, and we will discuss it at the video conference next week.” You needed to submit the documents after the conference, and then you sorted them out and sent them to the leaders for review. The leader replied, " Good job. The contents are comprehensive and clear. I've just sent revised opinions to you. You can upload the file after modification."

中文版本：

主动网络无礼情境:

你在一家公司上班，日常工作主要通过线上沟通。近段时间，你跟直属领导的交流情景如下。领导安排工作时不说清楚要求，让你直接做，做完之后又说：“缺东少西的，不行回家歇着吧！”后来项目遇到问题，你汇报了项目停滞的原因以及项目弊端，希望调整方案，但领导回复你：“我不知道你能干什么，动动脑子好吗?”由于想法不一致，你和领导发生了争执，领导在工作群里含沙射影地说你不服从管理，阴阳怪气地说：“有些人待在公司真是屈才了!”

被动网络无礼情境:

你在一家公司上班，日常工作主要通过线上沟通。近段时间，你跟直属领导的交流情景如下。请示工作时总是得不到有效回复，各种被敷衍或拖延。客户有疑问，但你也不能确定，于是去问领导，而领导只回复了“哦”，没回答问题。你马上追问，却没有得到任何回复，你只能不停地跟客户道歉。昨天上报文件，你发给领导审阅，明明显示领导“已读”，却一直不回复你，打电话也不接。但在同一时间段，领导在工作群里转发了公司的通讯推送。

控制组情境:

你在一家公司上班，日常工作主要通过线上沟通。近段时间，你跟直属领导的交流情景如下。安排工作时领导往往会发送明确的要求，有疑问时也会第一时间解答。上周项目推进遇到问题，你汇报了项目停滞的原因以及项目弊端，希望能调整方案，领导回复你：“反应及时，做得不错，做一个调整方案，下周视频会议上集中讨论。”讨论后要上报文件，你整理好发给领导审阅，领导读后回复说：“很好，全面清晰。刚才发了修改意见，再完善一下，可以交了。”
